# Abusive Sexting in Adolescence: Prevalence and Characteristics of Abusers and Victims

**DOI:** 10.3389/fpsyg.2021.610474

**Published:** 2021-02-24

**Authors:** Ricardo Barroso, Eduarda Ramião, Patrícia Figueiredo, Alexandra M. Araújo

**Affiliations:** ^1^Department of Education and Psychology, University of Trás-os-Montes and Alto Douro, Vila Real, Portugal; ^2^Faculty of Psychology and Education Sciences, University of Porto, Porto, Portugal; ^3^Department of Psychology and Education, Portucalense University, Porto, Portugal

**Keywords:** sexting behavior, violence, adolescence, sexual behavior–psychology, sexuality, cyber abuse

## Abstract

Sexting has been defined as sending, receiving, or forwarding sexually explicit messages, images, or photos to others through digital platforms, and can assume more consensual or more abusive and violent forms. This study aims to explore the prevalence of abusive sexting in Portuguese adolescents and the psychological characteristics of sexting abusers in terms of emotional and behavioral problems, potential markers of psychopathy, childhood trauma and maltreatment, and different forms of aggression. A cross-sectional study was conducted with 4,281 participants, aged 12–20 years (2,264 girls and 2,017 boys), of whom 204 (4.8%) engaged in abusive sexting behaviors and 182 (4.3%) self-identified as being a non-consensual sexting victim. Abusive sexting was more common among boys and middle adolescents, and abusive sexting victims were more likely to be children of single-parent families. Engaging in abusive sexting and being a victim of abusive sexting were also related to behavioral and emotional problems, callousness, experiences of neglect and abuse in childhood, and various forms of aggression. Implications for future research and intervention are discussed.

## Introduction

Nowadays, adolescents use personal technological devices for all types of social interactions, including sexual exploration and behavior. An adolescent practice of receiving public attention is the self-production of sexual images. Sexting can be described as sending or publishing sexually provocative text messages and images, including nude or half-nude photographs or videos, *via* mobile phones or the Internet (Mitchell et al., 2012; Cooper et al., 2016; Alonso and Romero, 2019). Sexting can also include receiving sexual texts and images of others, or exploiting image content or sexting behavior of others, by forwarding or sharing images (Drouin et al., 2013; Klettke et al., 2014; Cooper et al., 2016).

This practice can be seen as a normal and contemporary form of sexual expression and intimate communication within romantic and sexual relationships (Cooper et al., 2016; Englander, 2019; Barroso et al., 2020c), but also as a way of self-expression, exploration, and establishment of identity (Dir et al., 2013). In addition, images can be taken between friends, for example, as a joke (Cooper et al., 2016), or to increase popularity and acceptance within the peer group (Abeele et al., 2014). Some studies have observed that sexting is not always associated with other types of sexual harassment (Ross et al., 2019). While some studies report similar rates of self-producing and sending sexual images between genders (Dake et al., 2012; Rice et al., 2012), others have found different prevalence rates for girls and boys, with either boys being more likely to engage in sexually revealing self-exposures (Jonsson et al., 2014), or girls engaging more in sexting behaviors (Mitchell et al., 2012; Reyns et al., 2013; Martinez-Prather and Vandiver, 2014; Gregg et al., 2018).

If, on the one hand, sexting can be seen as a normative, consensual component of the exploration of sexuality during adolescence, on the other hand it may constitute a behavior of aggression and violence associated with various problems, such as risky sexual behavior or an increased likelihood of online victimization (Gámez-Guadix et al., 2017; Gámez-Guadix and de Santisteban, 2018; Marengo et al., 2019; Longobardi et al., 2020). The way the text message is performed and the use of sexual images can determine the legality of the adolescent’s behavior. Sexual abuse may occur based on the dissemination of photos or videos of a person without their consent, and/or by forcefully exposing a person to sexual material, by, for example, forcing someone to watch movies or videos of people having sex (Barroso et al., 2020a,b). From a conceptual point of view, what technically defines the presence or absence of sexual violence, as well as the nature of the interaction and relationship in question, is consent, equality, and coercion (see Rich, 2003; Barroso, 2016). Therefore, consensual sexting (voluntarily sending sexual content) has been distinguished from non-consensual or abusive sexting (when an image is incorrectly used and sent without permission), being the latter a form of sexual violence (Walker et al., 2011; Alonso and Romero, 2019).

Given the heterogeneous nature of sexting, it is important to understand the different motivations that may underpin different sexting behaviors (Strohmaier et al., 2014; Bianchi et al., 2016). Sexting motivations related to sexual and social goals are more often listed, and experimental sexting sources are commonly considered (Drouin and Tobin, 2014; Walrave et al., 2015; Bianchi et al., 2016, 2017). Lee et al. (2016) suggest the most common reason to participate in sexting is related to peer pressure and coercion, specifically among girls. Higher social competence has been described as negatively related to engaging in all sexting behaviors, but particularly more for receiving and forwarding than for sending and receiving, and especially more for girls than for boys (Casas et al., 2019). Additionally, negative outcomes of sexting are much more common among certain groups, namely young teenagers or pre-teenagers, and those who experience negative pressure or coercion to sext, especially outside an established relationship (Rice et al., 2014). Recently, a study from Van Ouytsel et al. (2019) showed that sexual minority adolescents are more likely to experience, but not to perpetrate, abusive sexting behaviors. In line with this, the Cooper et al. (2016) review about sexting in adolescents identified four principal motivations for sexting, namely, (a) flirting and trying to get the romantic attention of a potential partner (Dir et al., 2013; Temple and Choi, 2014), (b) expressing normal sexuality within a dating relationship (Renfrow and Rollo, 2014), (c) experimenting with sexuality and identity (Chalfen, 2009; O’Sullivan, 2014), and (d) responding to pressure from one’s partner or friends to conform to perceived normal behavior in the peer group (Walrave et al., 2014).

Identifying the factors that facilitate sexting among adolescents will help to achieve a better understanding of sexting in general, and of abusive sexting in particular, and also why adolescents engage in such risky behaviors. Among the studied correlates of sexting (Abeele et al., 2014; Hudson and Marshall, 2017), research has identified the influence of engaging in other sexual behaviors (Smith et al., 2014), the will to sext (van Oosten et al., 2017), a perceived need to seek popularity (Abeele et al., 2014), participating in cybergossip (Ringrose et al., 2013), and social competence (Bauman, 2015). Certain personality traits also seem to increase the likelihood of engaging in sexting: although most studies in this field were conducted with university students (see Whiteside and Lynam, 2001; Saulsman and Page, 2004; Ferguson, 2011; Settles et al., 2012; Delevi and Weisskirch, 2013; Dir et al., 2013), studies with adolescents showed relations between sexting and higher levels of sensation seeking (Van Ouytsel et al., 2014), as well as impulsivity (Temple et al., 2014). Similarly, a longitudinal study (Alonso and Romero, 2019) with adolescents from 12 to 19 years showed that adolescents who practice sexting are more likely to score higher on depression, impulsivity, and vulnerability. Thus, it is possible that adolescents who are more emotionally vulnerable may use sexting as a way to gain acceptance from their peers. In addition, the inability to control impulses can contribute to sending messages, photos and videos, without considering the possible consequences.

Internet addiction problems have been related to perceived maternal availability, cognitive reappraisal, and callousness traits (Trumello et al., 2018). In addition, some authors have shown interest in the study of factors associated with sexting in several dimensions such as family communication (Bianchi et al., 2019) and personality traits (e.g., honesty-humility, conscientiousness, emotionality, and extraversion; Morelli et al., 2020). However, there are few studies about factors that influence abusive sexting, namely if these factors may be associated with childhood traumas, psychopathic traits, or emotional problems.

The current study is innovative in this field, as it explores specifically abusive sexting, both in the perspective of the abuser and the victim, respectively, when the adolescent sends sexual pictures of another person, without their consent, or experiences cybervictimization because sexually explicit pictures or videos of themself were shared online with other people without consent. Accordingly, the purpose of the current study was to examine the prevalence and demographics of abusive sexting behaviors and abusive sexting victimization in a wide sample of Portuguese adolescents, and to explore the associations between abusive sexting and a set of psychological adjustment variables related to antisocial behavior and/or aggressive behavior perpetration (Moffitt, 1993; Loeber et al., 2008; Farrington, 2009). Sexting abusers are compared to adolescents who have not participated in such behaviors, and victims are compared with peers who have not had such an experience, in terms of emotional and behavioral problems, potential markers of psychopathy, childhood trauma and maltreatment, and different forms of aggression. The examination of links between abusive sexting and personal characteristics may provide crucial information for the identification of at-risk youth and the provision of timely and targeted prevention interventions.

## Method

### Participants

Data were drawn from the Interpersonal Violence Prevention Program (PREVINT)^1^ (Barroso et al., 2018). PREVINT is an original psychological intervention program designed to prevent the development and expression of aggression in adolescence. Previously to the intervention process, the project collected data from of 4,281 youth (ages 12−20; *M*_*age*_ = 14.51, *SD* = 1.83; 2,017 boys and 2,264 girls) attending 52 public middle- and high-schools, in rural and urban areas, from various districts of the country, both mainland and islands. Roughly half of the participants came from working-class families (51%). According to the developmental stages of adolescence and young adulthood (Steinberg and Morris, 2001; Johnson et al., 2009), participants were classified as: early adolescents (12–13 years, *n* = 1,449, 33.8%), middle adolescents (14–16 years, *n* = 2060, 48.1%), and late adolescents (17–20 years, *n* = 772, 18.0%). Regarding their socioeconomic status, they were classified as being from working-class families (*n* = 1,217, 28.4%) or middle- or upper-class families (*n* = 3064, 71.6%). Most were children of married (or the legal equivalent) couples (*n* = 3,097, 72.3%), while the others were children of divorced (or the legal equivalent) or widowed single-parent households (*n* = 1,184, 27.7%).

## Measures

### Social Desirability Scale

Social desirability scale (EDS-20; Almiro et al., 2016) is a self-report measure, with 20 items of dichotomous response (yes/no), validated for the age groups in this sample. Example items include “*Have you ever detested someone?*” and “*Have you ever taken advantage of someone?*” Prior to data analyses, all participants were screened for social desirability, ruling out adolescents who scored over *M* = 14.73, as they showed a tendency to transmit socially desirable responses rather than choosing responses that are a true reflection of their behaviors or feelings (Grimm, 2010). Cronbach’s alpha for total scale was.80.

### Abusive Sexting

Adolescents were asked two questions regarding sexting behaviors: (a) abusive sexting: “*Have you ever shared sexually explicit images or videos of other people without their consent?*”; and (b) abusive sexting victimization: “*Have sexually explicit images or videos of yourself ever been shared with other people without your consent?.*” Answers were rated as 0 = No, 1 = Yes.

### Youth Self Report

Youth self report (YSR; Achenbach and Rescorla, 2001; Portuguese version: Gonçalves et al., 2007). The YSR is a self-report questionnaire designed for school-age children and adolescents (ages 11−18) to obtain self-ratings of emotional, behavioral, and social problems. Items are rated on a three-point scale (0 = not true, 1 = somewhat or sometimes true, and 2 = very true or often true), based on the preceding 6 months. In this study, the syndromes Anxious/Depression (13 items; e.g., “*I feel worthless or inferior*”; α = 0.85), Social Problems (11 items; e.g., “*I don’t get along with other kids*”; α = 0.78), Opposition (Rule Breaking) Behavior (16 items; e.g., “*I cut classes or skip school*”; α = 0.60), and Aggressive Behavior (17 items; e.g., “*I destroy things belonging to others*”; α = 0.84) were used. According to the criteria suggested by Ponterotto and Ruckdeschel (2007) regarding the adequacy of internal consistency measures, and considering the marginal alpha value of Opposition Behavior subscale (α = 0.60) and the number of items (16 items), we decided to remove this scale from statistical analysis.

### Inventory of Callous-Unemotional Traits

Inventory of callous-unemotional traits [ICU; Essau et al., 2006; Portuguese version by Pechorro et al. (2014)]. The ICU is a questionnaire designed to assess callous and unemotional traits on a 4-point scale ranging from 0 (“Not at all true”) to 3 (“Definitely true”). This measure has been used with clinical and community samples of youth ranging from early adolescence to late adolescence/emerging adulthood (age range = 12–20 years). Three subscales were used: Uncaring (eight items; e.g., “*I hide my feelings from others*”; α = 0.86), Callousness (11 items; e.g., “*I do not care who I hurt to get what I want*”; α = 0.72), and Unemotional (five items; “*I feel bad or guilty when I do something wrong”*; α = 0.43). Although a low value of alpha could be due to a low number of questions (Nunnally, 1978), we decided to remove this scale from comparative statistical analysis.

### Childhood Trauma Questionnaire

Childhood trauma questionnaire [CTQ; Bernstein et al., 2003; Portuguese version by Dias et al. (2013)]. The CTQ is a 28-item questionnaire aimed to quantify self-reported childhood trauma history in adolescent and adult populations (from 12 years old). Responses are measured on a 5-point Likert scale form 1 (“Never true”) to 5 (“Very often true”). Childhood trauma was measured using five subscales: Emotional abuse (e.g., “*I thought that my parents wished I had never been born*”; α = 0.81), Emotional neglect (e.g., “*I felt loved*”; α = 0.82), Sexual abuse (e.g., “*I believe that I was sexually abused*”; α = 0.89), Physical abuse (e.g., “*I believe that I was physically abused*”; α = 0.83), and Physical neglect (e.g., “*I don’t have enough to eat*”; α = 0.60). Each subscale contains five items, and an additional three items are intended to measure any tendency to minimize or deny the abuse.

### Reactive-Proactive Aggression Questionnaire

Reactive-proactive aggression questionnaire (RPQ; Raine et al., 2006; Portuguese version by Pechorro et al., 2017). The RPQ is a 23-item self-report measure that distinguishes between reactive and proactive aggression, with items scored on a frequency scale ranging from never (Score = 0) to often (Score = 2). Reactive aggression is characterized by high emotional activation, impulsivity, and hostility (e.g., “*Reacted angrily when provoked by others*”), and proactive aggression is characterized by a tendency toward instrumental, planned, non-empathetic, and cold strategy behavior (e.g., “*Had fights with others to show who was on top*”). Cronbach’s alphas are.92 for the proactive subscale and.83 for the reactive subscale. This instrument has been used with samples of youth (ages 6–18 years), and adults (ages above 18 years).

### Buss–Perry Aggression Questionnaire-Short Form

Buss–Perry aggression questionnaire-short form [BPAQ-SF; Buss and Perry, 1992; Bryant and Smith, 2001; Portuguese version by Pechorro et al. (2016)]. The BPAQ measures four aspects of human aggression, with 12 items scored on a 5-point scale (from 1 = “Extremely uncharacteristic of me” to 5 = “Extremely characteristic of me”). The scales are: Physical Aggressiveness (three items; e.g., “*There are people who pushed me so far that we came to blows*”; α = 0.77), Verbal Aggressiveness (three items; e.g., “*My friends say that I’m somewhat argumentative*”; α = 0.75), Anger (three items; e.g., “*I have trouble controlling my temper*”; α = 0.74), and Hostility (three items; e.g., “*Other people always seem to get the breaks*”; α = 0.79). This instrument has been used with participants with ages of 11 years old and above.

### Procedures

The participants were students from Portuguese schools. In addition to the institutional authorization from the Portuguese Ministry of Education, all participants were informed of the goals of the study and the confidentiality and anonymity of their responses were guaranteed. The research protocol was approved by University of Trás-os-Montes and Alto Douro Ethics Committee. Written consent was collected from participants’ parents/legal guardians. Data were collected through computer-assisted self-reports on school computers (or smartphones, when authorized) during regular classes by using an Internet-based survey hosted on a secure institutional server. Participation in this research was voluntary and did not imply any monetary payment or delivery of material goods.

### Data Analyses

Frequencies, proportions, and chi-square tests were calculated to evaluate associations between engaging in abusive sexting and abusive sexting victimization, as well as differences by sex, age, and family background. Standardized residuals were analyzed to identify significant deviations of observed counts from expected frequencies. Student *t*-tests were calculated to analyze differences between sexting abusers and non-abusers and abusive sexting victims and non-victims. Cohen’s *d* statistics were calculated to determine effect sizes: with values of 0.20, 0.50, and 0.80 representing small, medium, and large effects, respectively (Cohen, 1988).

## Results

### Prevalence and Sociodemographic Factors

From a total of 4,281 participants who completed the questionnaires, 204 (4.8%) reported abusive sexting behaviors, i.e., sending unauthorized sexually explicit images or videos of others, and 182 (4.3%) self-identified as being an abusive sexting victim, i.e., sexually explicit images or videos of themselves were shared without their consent. There was a significant association between engaging in abusive sexting and being an abusive sexting victim, χ*^2^*(1) = 132.15, *p* < 0.001. Adolescents both engaged in and victims of abusive sexting were more frequent than expected (*n* = 41). In addition, there were less cases than expected in being a sexting abuser but not a victim (*n* = 163), as well as in being a victim, but not an abuser (*n* = 141).

Table 1 presents the associations between sociodemographic variables and abusive sexting behaviors, as well as abusive sexting victimization. A significant difference between the observed and expected frequency of abusive sexting behaviors was found in boys and girls: a higher number of sexting abusers was found for male adolescents (*n* = 143) than for female adolescents (*n* = 61). Although the number of abusive sexting victimization cases was higher for girls (*n* = 100) than for boys (*n* = 82), the association between gender and abusive sexting was not significant for victimization experiences.

**TABLE 1:** Prevalence of abusive sexting behaviors and abusive sexting victimization.

	Engaging in abusive sexting behaviors			Abusive sexting victimization		
			
	No	Yes	χ*^2^*	No	Yes	χ*^2^*
	*n* (%)	*n* (%)		*n* (%)	*n* (%)	
**Sex**						
Male	1,874(92.9)	143 (7.1)	45.41⁢***a	1,935(95.9)	82 (4.1)	0.32^*a*^
Female	2,203(97.3)	61 (2.7)		2,164(95.6)	100 (4.4)	
**Age**						
Early adolescents	1,403(96.8)	46 (3.2)	12.26⁢**b	1,397(96.4)	52 (3.6)	2.62^*b*^
(12–13 years)						
Middle adolescents	1,944(94.4)	116 (2.7)		1,963(95.3)	97 (4.7)	
(14–16 years)						
Late adolescents	730 (94.6)	42 (5.4)		739 (95.7)	33 (4.3)	
(17–20 years)						
**Family socioeconomic status**						
Working-class	1,156(95.0)	61 (5.0)	0.23^*a*^	1,155(94.9)	62 (5.1)	2.97^*a*^
Middle and upper-class	2,921(95.3)	143 (4.7)		2,944(96.1)	120 (3.9)	
**Parents’ marital status**						
Married or equivalent	2,951(95.3)	146 (4.7)	0.01^*a*^	2,983(96.3)	114 (3.7)	8.78⁢**a
Single-parent, divorced or equivalent, or widowed	1,113(95.2)	56 (4.8)		1,102(94.3)	67 (5.7)	

**p < 0.01, ***p < 0.001. ^*a*^df = 1, ^*b*^df = 2.

There was also a significant association between age and engaging in abusive sexting: early adolescents (12–13 years; *n* = 46) were less likely and middle adolescents (14–16 years; *n* = 116) were more likely to engage in abusive sexting behaviors. The inspection of standardized residuals indicated no significant difference between expected and counted frequencies of sexting abusers in late adolescents (*n* = 42). The number of abusive sexting victims was higher in middle adolescents (*n* = 97) than in early adolescents (*n* = 52) and late adolescents (*n* = 33). However, the association between age and abusive sexting victimization was not statistically significant.

No significant associations were found between family socioeconomic status and engaging in abusive sexting, nor abusive sexting victimization. Finally, no significant association was found between the parents’ marital status and being a sexting abuser, but the association was observed for being an abusive sexting victim. Children of single-parent families were more likely than expected (*n* = 67) to be a victim of sexting than children of married couples.

### Group Differences for Psychological Adjustment Variables

Systematic differences were found between adolescents who engaged in abusive sexting and those that did not, as shown in Table 2, for most of the studied psychological adjustment variables. When compared to those who did not engage in such behaviors, sexting abusers reported: significantly higher levels of aggressiveness, social problems, anxiety and depression; significantly more frequent experiences of emotional abuse, emotional neglect, sexual abuse, and physical neglect in childhood; significantly higher levels of callousness and lower levels of uncaring (but not unemotional) traits; significantly more frequent proactive and reactive aggression; and described themselves as significantly more physically and verbally aggressive, angry, and hostile. Such differences were also observed for the victims of abusive sexting, with the exception of uncaring traits, where no differences were found between groups. It is worthy to notice that the greatest differences between groups for abusive sexting was found in aggressiveness, proactive aggression, and physical aggression, and the least differences were found for traumatic experiences of emotional abuse in childhood, anxiety and depression, and anger. For abusive sexting victimization, differences were typically less expressive, compared to the ones observed for abusive sexting; nonetheless, the differences were higher in emotional abuse and sexual abuse, and lower in callous traits and anger.

**TABLE 2:** Group differences for psychological adjustment variables in abusive sexting behaviors and abusive sexting victimization.

	Engaging in abusive sexting behaviors				Abusive sexting victimization			
			
Psychological adjustment variables	No	Yes	*t*(3596)	Cohen’s	No	Yes	*t*(3596)	Cohen’s
	*M (SD)*	*M (SD)*		*d*	*M (SD)*	*M (SD)*		*d*
Aggressiveness (YSR)	9.10 (5.31)	12.23 (7.42)	−7.51***	0.49	9.13 (5.35)	11.91 (7.23)	−6.27***	0.44
Social problems (YSR)	4.64 (3.71)	6.37 (4.95)	−5.96***	0.40	4.64 (3.72)	6.49 (4.86)	−6.02***	0.43
Anxiety and Depression (YSR)	7.67 (5.17)	9.03 (6.34)	−3.38**	0.24	7.63 (5.17)	10.01 (6.20)	−5.60***	0.42
Emotional abuse (CTQ)	8.18 (3.83)	9.04 (4.48)	−2.91**	0.21	8.11 (3.74)	10.75 (5.48)	−8.46***	0.56
Emotional neglect (CTQ)	9.78 (4.73)	11.08 (5.55)	−3.56***	0.25	9.78 (4.75)	11.20 (5.25)	−3.65***	0.28
Sexual abuse (CTQ)	5.85 (2.55)	6.82 (3.92)	−4.77***	0.29	5.83 (2.53)	7.37 (4.17)	−7.18***	0.44
Physical neglect (CTQ)	6.81 (2.76)	8.25 (3.73)	−6.65***	0.44	6.83 (2.79)	7.93 (3.45)	−4.77***	0.35
Callousness (ICU)	11.56 (5.28)	14.13 (5.63)	−6.32***	0.47	11.64 (5.29)	12.72 (5.89)	−2.49*	0.19
Uncaring (ICU)	15.85 (5.52)	14.16 (5.23)	3.99***	0.32	15.80 (5.52)	15.00 (5.59)	1.77	0.14
Proactive aggression (RPQ)	3.42 (4.37)	7.14 (5.97)	−10.86***	0.71	3.52 (4.45)	5.54 (5.82)	−5.51***	0.39
Reactive aggression (RPQ)	7.25 (4.12)	9.44 (5.05)	−6.86***	0.48	7.30 (4.15)	8.63 (4.98)	−3.90***	0.29
Physical aggression (AQ)	4.90 (2.37)	6.55 (3.13)	−8.90***	0.59	4.94 (2.40)	5.92 (3.11)	−4.92***	0.35
Verbal aggression (AQ)	6.08 (2.60)	7.17 (3.23)	−5.43***	0.37	6.10 (2.60)	6.91 (3.05)	−3.82***	0.26
Anger (AQ)	6.93 (2.99)	7.72 (3.10)	−3.42**	0.26	6.94 (2.98)	7.77 (3.40)	−3.42**	0.26
Hostility (AQ)	7.25 (3.17)	8.28 (3.32)	−.24***	0.32	7.25 (3.17)	8.33 (3.32)	−4.17***	0.33

*p < 0.05, **p < 0.01, and ***p < 0.001.

## Discussion

The current study examined the prevalence and associations of engaging in abusive sexting and abusive sexting victimization and psychological adjustment variables in a sample of Portuguese adolescents. The prevalence of abusive sexting behaviors (4.8%) and victimization (4.3%) was lower than reported in prior studies (e.g., Gámez-Guadix et al., 2017; Gámez-Guadix and de Santisteban, 2018), which was expected as prior research focused on general and consensual forms of sexting. The prevalence in abusive sexting in boys and girls is not consistent with what has been known for general sexting (Casas et al., 2019), as in the current study boys were more likely than girls to engage in abusive sexting. These gender differences were also found in earlier studies, where sexting presented different patterns in girls and boys (Burén and Lunde, 2018; Casas et al., 2019). Family socioeconomic background was irrelevant for abusive sexting, but parental marital status was not, as the children from single-parent households were at a higher risk of being a victim of abusive sexting.

Abusive sexting was related to behavioral and emotional problems, which is consistent with prior research that identified mental health issues as correlates of sexting (Gámez-Guadix et al., 2017; Gámez-Guadix and de Santisteban, 2018). Prior studies suggested an association between sexting behaviors and personality characteristics, such as conscientiousness and extraversion (Temple and Choi, 2014; Gámez-Guadix and de Santisteban, 2018). Callous and unemotional traits were also related to abusive sexting. To our knowledge, this is the first study that has identified such a relation, suggesting that abusive sexting in adolescents may be related to personality-related variables, which are more stable and therefore of a worse prognosis for abusers. It is known that callous and unemotional traits are associated with aggression, bullying, and other antisocial behaviors (Ang and Goh, 2010). The association between sexting and such traits may be explained by a lack of empathy, not caring about others, and not feeling remorse.

In this study, abusive sexting was related to childhood experiences of physical, emotional, and sexual neglect and abuse, suggesting that such past experiences may shape adolescents’ proneness to disregard interpersonal respect and trust. Previous research suggests that adolescents who engage in abusive sexting behavior have histories marked with more frequent physical and sexual abuse experiences (Jonsson et al., 2014). Yoder et al. (2018) suggested that youth exposed to violence or adversity in their homes may engage in sexting because they are in an emotionally disinhibited state.

Finally, abusive sexting was related to various forms of aggression, including reactive and proactive aggression, and hostility, anger, and physical and verbal aggression. This finding validates the suggestion that abusive sexting can be considered as an expression of aggressive behavior, and that it will probably co-occur with other manifestations of aggression in adolescence. In other words, these results suggest that the psychological characteristics presented by these young abusers could be explained as another manifestation of general antisocial tendencies. Prior research has already shown an association between verbal aggression and physical aggression (Beckmann et al., 2017), and that physical aggression specifically in dating violence may be related to sexting (Dake et al., 2012). According to Englander (2012), adolescents in abusive sexting reported more often histories of dating violence in high-school, compared to both youth who sexted in the absence of coercion and those who did not sext at all.

Taken altogether, these findings suggest that sexting abusers are at a higher risk of emotional and behavioral problems. However, this is not only the case for abusers, but also for victims. The current study found that victims of abusive sexting also presented higher levels of intra- and interpersonal problems, also experienced more abuse and neglect in the past, and also engaged more frequently in aggressiveness, when compared to youth who did not have such an experience.

Albeit the contributions of this study, it is not without limitations. First, the cross-sectional design of this study limits inferences about causality. Future research could explore the temporal dynamics between abusive sexting and victimization and mental health issues, by employing longitudinal designs that follow participants from the transition from childhood to adolescence, and from adolescence into young adulthood. Second, this study relied solely on self-reported measures, which can lead to shared method variance and reporting bias. In the future, it would be valuable to include multi-informant data and observational or qualitative methods to strengthen our understanding of adolescents’ experiences of abusive sexting and victimization. Third, the current study did not control for factors which may impact the results, such as dating and sexual experience. Future studies could further explore if abusive sexting occurs in the context of a dating relationship or if it is related to online deviant behavior, such as catfishing (i.e., pretending to be another person), inappropriate use of shared images, or even online theft or hacking personal presence online. Fourth, although participants were screened for social desirability, it is not guaranteed that all participants were all straightforward with their experiences of engaging in abusive sexting behaviors and especially in victimization. A cross-validation of the findings could be valuable with a new dataset, exploring the stability of prevalence and personal variables linked to abusive sexting across different samples. Finally, the current study did not explore other forms of sexting, including sending or receiving sexually explicit texts, and how and with whom do the adolescents exchange sexts with (e.g., a group of peers, adults, posting on social media platforms). Such details of abusive sexting could be explored in future studies.

Despite the limitations that may be identified, the current study contributes to research that attempts to describe and analyze abusive sexting in adolescence, by exploring the experiences of adolescents from a South-European country, and focusing on a particularly relevant type of sexting with close relations to cybervictimization. The findings suggest that abusive sexting, although not as widespread as more consensual forms of sexting, warrants further attention and research, as it is a damaging experience for adolescents’ psychological adjustment, both for abusers and for victims. The present study has important implications for clinicians and counselors in an intervention process, particularly concerning childhood experiences of abuse and neglect, associated with abusive sexting behaviors and especially with victimization.

## Data Availability Statement

The raw data supporting the conclusions of this article will be made available by the authors, without undue reservation.

## Ethics Statement

The studies involving human participants were reviewed and approved by Universidade de Trás-os-Montes and Alto Douro. Written informed consent to participate in this study was provided by the participants’ legal guardian/next of kin.

## Author Contributions

All authors listed have made a substantial, direct and intellectual contribution to the work, and approved it for publication.

## Conflict of Interest

The authors declare that the research was conducted in the absence of any commercial or financial relationships that could be construed as a potential conflict of interest.

## Footnotes

1 https://www.prevint.pt/en
